# The Role of Alveolar Epithelial Type II-Like Cells in Uptake of Structurally Different Antigens and in Polarisation of Local Immune Responses

**DOI:** 10.1371/journal.pone.0124777

**Published:** 2015-04-20

**Authors:** Johnnie Akgün, Irma Schabussova, Martin Schwarzer, Hana Kozakova, Michael Kundi, Ursula Wiedermann

**Affiliations:** 1 Institute of Specific Prophylaxis and Tropical Medicine, Centre for Pathophysiology, Infectiology and Immunology, Medical University of Vienna, Vienna, Austria; 2 Laboratory of Gnotobiology, Institute of Microbiology, v.v.i., Academy of Sciences of the Czech Republic, Novy Hradek, Czech Republic; 3 Institute of Environmental Health, Medical University of Vienna, Vienna, Austria; 4 Department of Rheumatology and Inflammation Research, Institute of Medicine, University of Göteborg, Göteborg, Sweden; University of Giessen Lung Center, GERMANY

## Abstract

**Background:**

Our previous studies on intranasal tolerance induction demonstrated reduction of allergic responses with different allergen constructs. The underlying mechanisms varied depending on their conformation or size.

**Objective:**

The aim of the present study was to compare the uptake of two structurally different allergen molecules within the respiratory tract following intranasal application.

**Methods:**

The three-dimensional Bet v 1 (Bv1-Protein) and the T cell epitope peptide of Bet v 1 (Bv1-Peptide) were labelled with 5,6-Carboxyfluorescein (FAM) and their uptake was investigated in lung cells and cells of the nasal associated lymphoid tissue from naive and sensitised BALB/c mice. Phenotypic characterisation of FAM^+^ lung cells after antigen incubation *in vitro* and after intranasal application was performed by flow cytometry. Impact of Bv1-Protein and Bv1-Peptide on cytokine profiles and gene expression *in vivo* or in an alveolar epithelial type II (ATII) cell line were assessed in mono- and co-cultures with monocytes using ELISA and quantitative real-time PCR.

**Results:**

Both antigens were taken up preferably by ATII-like cells (ATII-LCs) in naive mice, and by macrophages in sensitised mice. After intranasal application, Bv1-Peptide was taken up faster and more efficiently than Bv1-Protein. *In vivo* and *in vitro* experiments revealed that Bv1-Protein induced the transcription of thymic stromal lymphopoietin mRNA while Bv1-Peptide induced the transcription of IL-10 and MCP1 mRNA in ATII-LCs.

**Conclusion and Clinical Relevance:**

Both tested antigens were taken up by ATII-LCs under steady state conditions and induced different polarisation of the immune responses. These data may have an important impact for the generation of novel and more effective prophylactic or therapeutic tools targeting the respiratory mucosa.

## Introduction

Type I allergic disorders such as allergic rhinitis, asthma, and atopic eczema are affecting ~20% of westernised countries [1,2]. The initial contact site of inhaled allergens in the human body is the respiratory mucosa. In allergy-prone patients, this contact results in T helper type 2-skewed (T_H_2) immune response leading to IgE-mediated clinical manifestations. The only immune modifying and potentially curative treatment for type I allergy is allergen-specific immunotherapy (SIT). The goal of SIT is to generate a switch from allergen-specific T cells to tolerant or anergic T cells and down regulation of IgE-mediated immune responses in allergic patients [3]. Nevertheless, the use of natural allergen extracts in SIT is associated with certain drawbacks, such as varying allergen concentrations, presence of non-allergic materials, and *de novo* sensitisation against other components within the allergen extracts [4]. In order to increase the safety of SIT, we and others have generated recombinant allergens, non-IgE binding T cell epitopes, polypeptide constructs, or fragments of the allergens without IgE reactivity [5–8] and tested their efficacy in different mouse models of type I allergy [4].

In a mouse model of birch pollen allergy, intranasal administration of major birch pollen allergen Bet v 1 led to suppression of allergic immune responses and airway inflammation in sensitised mice [5,6]. Furthermore, we have shown that it is possible to induce tolerance by mucosal application of different allergen-derived peptides [7,8].

The precise mechanism of interaction between structurally diverse antigens and the respiratory epithelium, which might lead to sensitization or tolerance, is still far from being elucidated. Only recently, there is increasing evidence stressing the role of epithelial cells in orchestrating immune responses to allergens [9,10].

Upon contact with a pathogen, alveolar epithelial type II (ATII) cells can secrete antimicrobial proteins [11,12], components of the complement system [13], and a variety of cytokines and chemokines (e.g. IL-6, IL-8, IL-10, and MCP1) [11,14] which are involved in the recruitment of neutrophils, eosinophils, monocytes, T cells, and dendritic cells (DCs) to the alveolar region [15–20]. However, less is known about their function upon contact with allergens or modified allergen molecules.

In the present study, we aimed to investigate how differences in conformation of antigens (three-dimensional vs. linear) are influencing their recognition and uptake by innate immune cells and what is the consequence of this interaction in the context of steady state condition or allergy. As model antigens we used the three-dimensional recombinant Bet v 1 (Bv1-Protein) and the single T cell epitope peptide of Bet v 1 (Bv1-Peptide) and investigated their uptake by different cell populations *in vitro* and *in vivo*.

## Materials and Methods

### Mice

6- to 8-week-old female BALB/c mice were obtained from Charles River (Sulzfeld, Germany). Mice were housed under specific pathogen-free conditions at the Medical University of Vienna, Vienna, Austria. All experiments were approved by the Animal Ethics Committee of the Medical University of Vienna (BMWF-66.009/0193-II/3b/2013) and by the Austrian Federal Ministry of Science and Research.

### Antigens

Recombinant Bv1-Protein was obtained from Biomay AG (Vienna, Austria). Bv1-Peptide was synthesised and purified to >90–95% purity by preparative HPLC by piCHEM (Graz, Austria). Amino acid sequence of the Bv1-Peptide was as following: MGETLLRAVESY. For flow cytometry and confocal laser microscopy, the Bv1-Protein and the Bv1-Peptide were labelled with 5,6-Carboxyfluorescein (FAM) by piCHEM. The endotoxin content of Bv1-Protein and Bv1-Peptide was determined by the Limulus amoebocyte lysate (LAL) assay (Lonza, Basel, Switzerland). The levels of endotoxin were below 0.004 EU/μg in both antigens.

### Incubation of lung cells with antigens *in vitro*

Lungs from naive BALB/c mice were digested in RPMI containing 0.4 mg/ml collagenase II (Sigma-Aldrich, St. Louis, MO, USA) and 0.04 mg/ml DNase I (Sigma-Aldrich) for 1 hour at 37°C. Tissue was passed through a 70 μm cell strainer. Lung cells were stimulated with 5 μg/ml of Bv1-Protein-FAM or Bv1-Peptide-FAM for 0.5, 1, 6, 24, and 48 hours. FAM^+^ cells were detected by flow cytometry.

### Intranasal application of antigens

Naive and allergic mice were anesthetised with Sevofluran (Abbott, Vienna, Austria) and 20 μg of Bv1-Protein-FAM or Bv1-Peptide-FAM were applied intranasally. At different time points after the application (1, 6, 24, or 48 hours), nasal associated lymphoid tissue (NALT) and lungs were collected. FAM^+^ cells were detected by flow cytometry (FACSCalibur; BD Biosciences, San Jose, CA, USA).

### Culture conditions for A549 and THP1 cell lines

Human ATII cell line A549 (ATCC, Manassas, Virginia, USA) was cultures in DMEM with 10% heat-inactivated FCS (PAA, Cambridge, UK) and 5 mM L-glutamine (PAA). A549 cells were plated in 48 wells flat-bottom plates (5x10^5^ cells/well) and incubated until confluence. After removal of the culture medium, cells were incubated with 5 μg/ml of Bv1-Protein-FAM or Bv1-Peptide-FAM in DMEM with 10% FCS and 5 mM L-glutamine for different time periods (0.5, 1, 4, 24, and 48 hours). For confocal laser microscopy, 4x10^4^ cells incubated with Bv1-Protein or Bv1-Peptide were cytospined onto glass slides, fixed with ice-cold aceton, and mounted with DAPI mounting medium (Vectashield, CA, USA). Internalisation of Bv1-Protein-FAM and Bv1-Peptide-FAM was documented using the Confocal Zeiss LSM700 microscope (Zeiss, Göttingen, Germany).

THP1 monocyte/macrophage cell line (ATCC) was cultured in RPMI supplemented with 10% heat-inactivated FCS and 10 mM β-2 Mercaptoethanol (Carl Roth GmbH, Karlsruhe, Germany). Both cell lines were maintained at 37°C under a 5% CO_2_ atmosphere. For co-culture, A549 cells were plated in 48 wells flat-bottom plates (5x10^5^ cell/well) and incubated until confluence. THP1 cells were added in a concentration of 5x10^5^ cells/well. For transwell co-culture, the cells were separated with 0.4-μm-pore filters (Corning, NY, USA). The co-cultures were incubated with 5 μg/ml of the Bv1-Protein or the Bv1-Peptide for 6 days. Cells were harvested separately to evaluate gene expression and cell free supernatants were stores at -20°C for further analysis.

### Mouse model of Bet v 1 sensitisation and airway challenge

Allergic airway inflammation was performed as previously described [21]. Briefly, mice were sensitised three times intraperitonealy (on day 0, 14 and 28) with 1 μg of recombinant Bet v 1 (Biomay, Vienna, Austria) precipitated with 100 μl aluminium hydroxide (Alum, Serva, Heidelberg, Germany). Seven days after the last treatment, mice were challenged intranasally three times (day 35, 36 and 37) with 100 μg of birch pollen extract (Allergon, Välinge, Sweden). 20 μg of Bv1-Protein-FAM or Bv1-Peptide-FAM were applied intranasally at day 40. NALT and lungs were excised 1 or 6 hours later.

### Flow Cytometry

Anti-mouse B220, CD11b, CD11c, CD19, CD31, CD45, CD16/CD32, F4/80, and MHC class II (MHCII) mAbs were purchased from eBioscience (San Diego, CA, USA). Appropriate isotype-matched control antibodies were used to determine nonspecific staining. ATII-like cells (ATII-LCs) (CD11b^-^/CD11c^-^/CD16/32^-^/CD19^-^/CD31^-^/CD45^-^/F4/80^-^MHCII^+^), macrophages (CD11b^+^/CD11c^-^), DCs (CD11b^-^/CD11c^+^), and B cells (B220^+^/CD19^+^) were stained using standard procedures and analysed by flow cytometry.

### ELISA

Levels of IL-6 were measured by commercially available ELISA (Diaclone, Besancon, Cedex, France). IL-8 levels were measured in 96-well plates coated with 2 μg/ml of mouse anti-human IL-8 mAb (Thermo Scientific). 50 μl of a 1:10 dilution of the samples were investigated in triplicates. Thereafter 0.2 μg/ml of biotin-labelled mouse anti-human IL-8 mAb (Thermo Scientific) was applied followed by horseradish peroxidase-conjugated streptavidin. With tetramethylbenzidin substrate (Millipore, Billerica, MA, USA) colour development was achieved and the absorbance at 450 nm was measured.

### Isolation of ATII-LCs

Primary ATII-LCs were isolated as described by Driscoll B. *et al*. 2012 [22]. Briefly, mice were euthanized and left kidney was excised. Lungs were perfused with 5 ml of PBS through the right ventricle of the heart. One ml collagenase (Sigma-Aldrich) was instilled into the lungs via tracheal catheter followed by 0.5 ml 1% low-melt agarose (Sigma-Aldrich) warmed to 45°C. Lungs were immediately covered with ice for 2 minutes. Lungs were then dissected, placed in a culture tube containing 1 ml of collagenase (Sigma-Aldrich), and incubated for 45 minutes at room temperature. Lung tissue was teased apart, transferred to a culture dish containing 7 ml of DMEM and 100 μl of DNase (Sigma-Aldrich) and then filtered through 100-, 40- and 25 μm nylon meshes. Single cells were stained with a mixture of APC labelled anti-mouse antibodies (CD11b, CD11c, CD16/32, CD19, CD31, CD45, and F4/80). ATII-LCs were isolated by negative selection with anti-APC coated magnetic beads using the Magnetic Activated Cell Sorting (MACS) system (Miltenyi Biotec, Bergisch Gladbach, Germany) according to the manufacturer’s instructions.

### Quantification of mRNA

200 ng of RNA was reversed transcribed using a cDNA kit (Bio-Rad Laboratories, Hercules, CA, USA) according to the manufacturer's instructions. Primers used for quantitative real-time PCR reactions had the following sequences: human β-actin forward: 5’-GGACTTCGAGCAAGAGATGG-3’ and reverse: 5’-AGCACTGTGTTGGCGTACAG-3’; human IL-10 forward: 5’-AGAACAGCTGCACCCACTTC-3’ and reverse: 5’-GCATCACCTCCTCCAGGTAA-3’; human MCP1 forward: 5’-CCCCAGTCACCTGCTGTTAT-3’ and reverse: 5’-TGGAATCCTGAACCCACTTC-3’; human thymic stromal lymphopoietin (TSLP) forward: 5’-GAGTGGGACCAAAAGTACCG-3’ and reverse: 5’-TGGGCACCAGATAGCTAAGG-3’; murine β-actin forward: 5’-GCTCTTTTCCAGCCTTCCTT-3’ and reverse: 5’-CTTCTGCATCCTGTCAGCAA-3’; murine IL-10 forward: 5’-CCAAGCCTTATCGGAAATGA-3’ and reverse: 5’-TTTTCACAGGGGAGAAATCG-3’; murine MCP1 forward: 5’-AGGTCCCTGTCATGCTTCTG-3’ and reverse: 5’-TCTGGACCCATTCCTTCTTG-3’; murine TSLP forward: 5’-CGGATGGGGCTAACTTACAA-3’ and reverse: 5’-AAATGTTTTGTCGGGGAGTG-3’. Relative gene expression was quantified using the 2^−ΔΔCT^ method. Expression of IL-10, MCP1 and TSLP was normalised to expression of the housekeeping gene β-actin and relative gene quantification was performed by comparing Bv1-Protein or Bv1-Peptide stimulated samples with control samples.

### Statistics

Statistical analysis was performed with GraphPad Prism 5 software (San Diego, CA, USA). Data were assessed for significance using the Student’s *t* test (for comparison of two groups) or ANOVA (for multiple group comparison). For 2^−ΔΔCT^ method values were log transformed to obtain a symmetric distribution. These values were analysed by a General Linear Model with Bv1-Protein/Bv1-Peptide as the main factor of interest and mice nested within the experiment factor. Mice (3 per group) and experiment (1/2) were defined as random factors. By this procedure potential differences between experiments (different RT-PCR plates) and differences between animals for which triplicate measurements were available are accounted for in the analysis of the difference between reaction to Bv1-Protein and Bv1-Peptide. Homogeneity of variances was examined by Levene's tests and normality of residuals was checked by Kolmogorov-Smirnov tests with Lilliefors' corrected p-values. A similar approach was used for the experiments with cell lines. Experiments were repeated three times in this case and the analysis was performed applying a model with a random factor (experiments 1 to 3) and a fixed factor (Bv1-Protein/Bv1-Peptide).

## Results

### ATII-LCs from lungs of naive mice are the major population internalising Bv1-Protein and Bv1-Peptide *in vitro*

**Fig 1:**
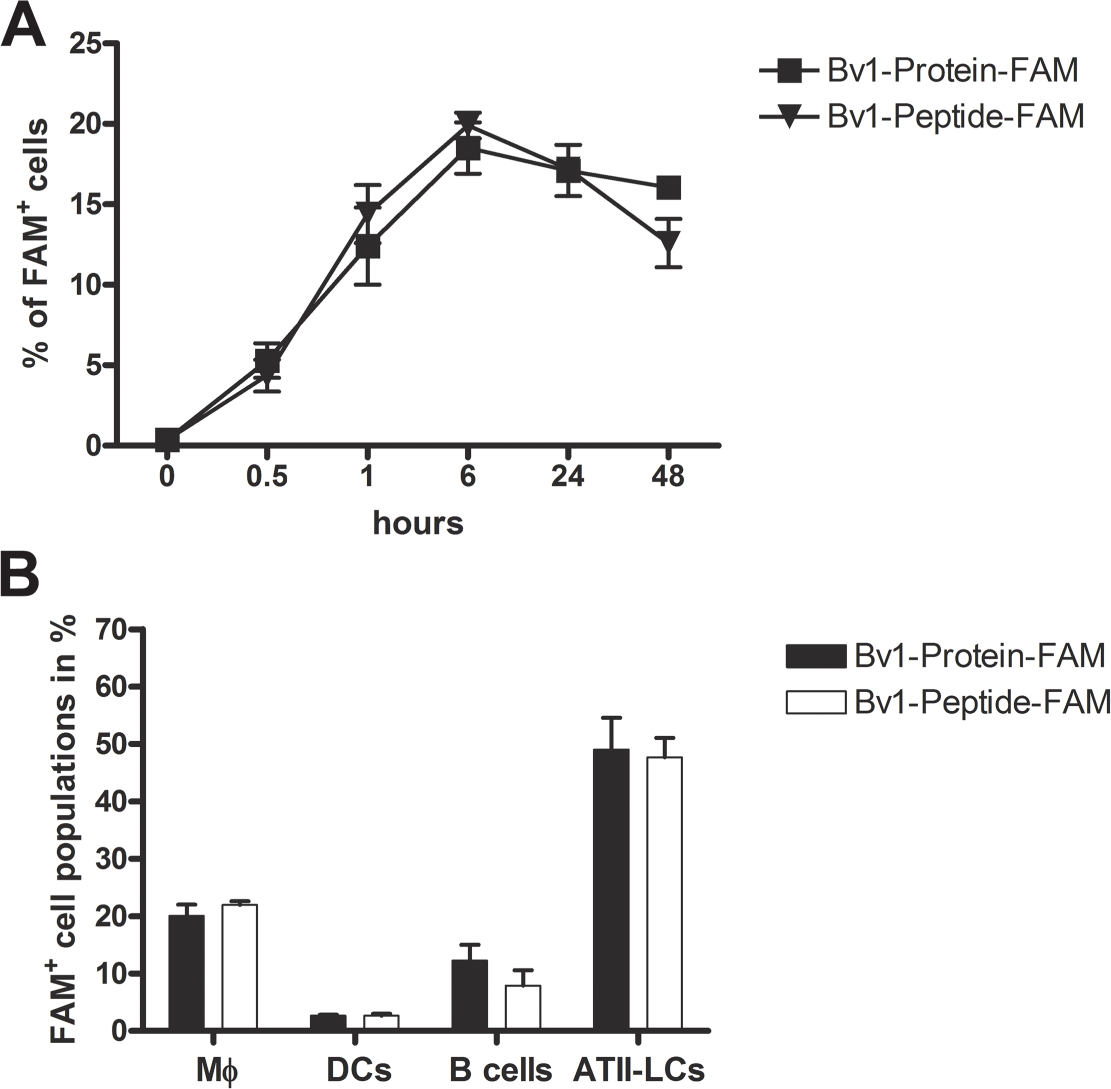
Time-dependent internalisation of Bv1-Protein and Bv1-Peptide in lung cells and phenotypic characterisation of FAM^+^ lung celIs *in vitro*. (**A**) Lung cells from naive BALB/c mice were incubated with 5 μg/ml Bv1-Protein-FAM or Bv1-Peptide-FAM for 0.5, 1, 6, 24, and 48 hours. Samples were analysed by flow cytometry. Dead cells were identified by 7-AAD staining and excluded from analysis. (**B**) FAM^**+**^ cells were gated and antigen uptake capacity of macrophages (CD11b^**+**^/CD11c^**-**^), dendritic cells (CD11b^**-**^/CD11c^**+**^), B cells (B220^**+**^/CD19^**+**^), and ATII-LCs (CD11b^**-**^/CD11c^**-**^/CD16/32^**-**^/CD19^**-**^/CD45^**-**^/F4/80^**-**^) was investigated after 6 hours. (**A** and **B**) Data are the pool from three independently performed experiments of identical design. Values represent means ± SEM. ATII-LCs = ATII-like cells; DCs = dendritic cells; Mϕ = macrophages.

Lung cells derived from naive BALB/c mice were incubated with 5 μg/ml of FAM-labelled Bv1-Protein or Bv1-Peptide for different time points (0.5–48 hours). Antigen internalisation reached the maximum after 6 hours of incubation (Fig 1A) and slightly decreased after 48 hours. In order to characterise the cell populations internalising Bv1-Protein and Bv1-Peptide, naive lung cell cultures were incubated for 6 hours with either of the antigens *in vitro*. ATII-LCs reveal to be the most efficient population in uptake of Bv1-Protein and Bv1-Peptide followed by macrophages, B cells, and DCs (Fig 1B).

### ATII-LCs in naive but not in sensitised mice are the major population taking up antigens

**Fig 2:**
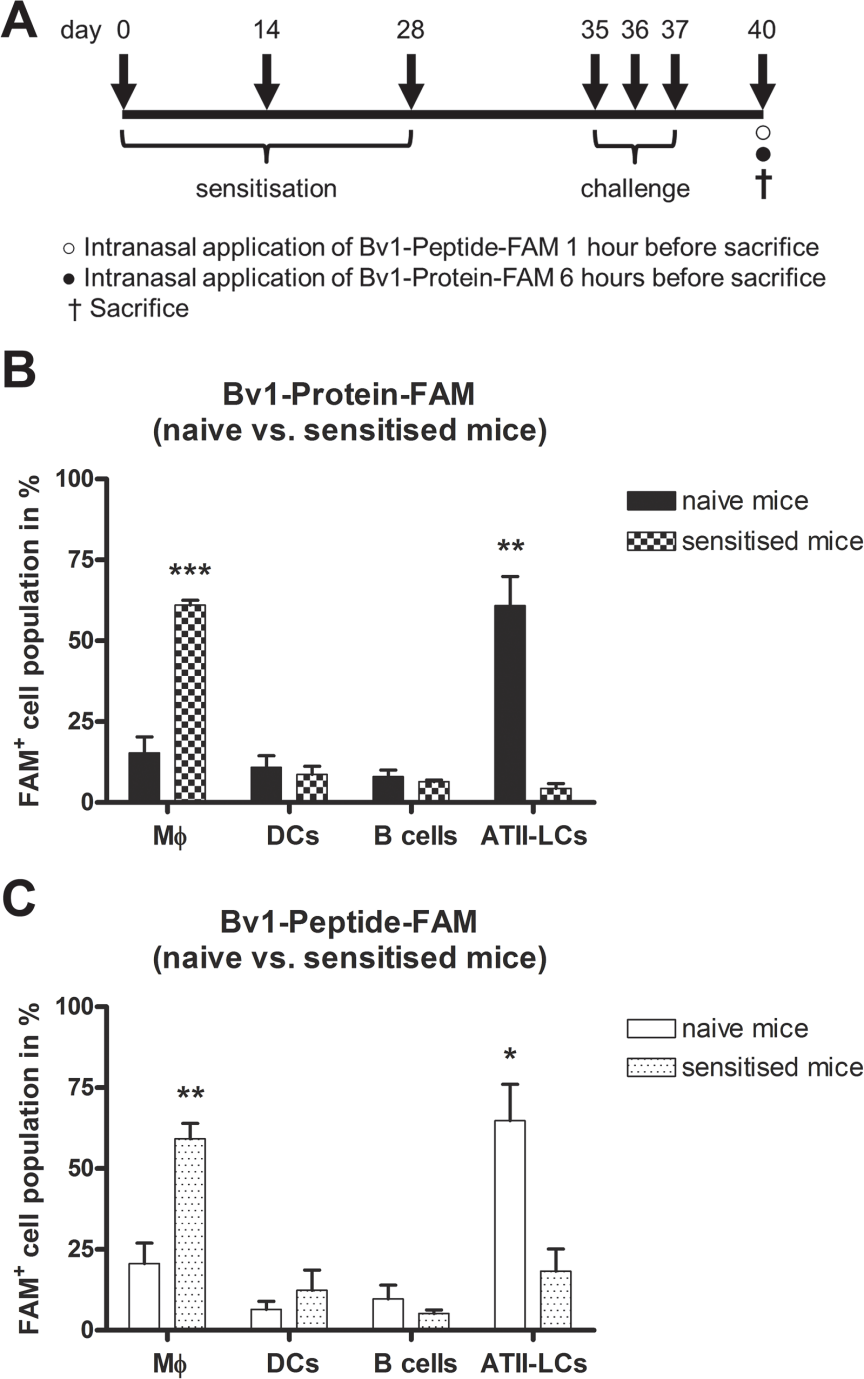
Uptake of Bv1-Protein and Bv1-Peptide in lungs of naive and sensitised mice *in vivo*. (**A**) Experimental design: Mice were sensitised 3 times intraperitonealy with 1 μg of Bet v 1 on days 0, 14 and 28. Intranasal challenge with 100 μg of birch pollen extract was performed one week after last sensitisation on days 35, 36 and 37. On day 40, 20 μg of Bv1-Protein-FAM and Bv1-Peptide-FAM were intranasal administered to naive and allergic BALB/c (n = 3) mice and lungs were collected after 1 (for Bv1-Peptide) or 6 hours (for Bv1-Protein). Dead cells were identified via 7-AAD staining and excluded from analysis. (**B** and **C**) FAM^**+**^ cells were gated and antigen uptake capacity of macrophages (CD11b^**+**^/CD11c^**-**^), dendritic cells (CD11b^**-**^/CD11c^**+**^), B cells (B220^**+**^/CD19^**+**^), and ATII-LCs (CD11b^**-**^/CD11c^**-**^/CD16/32^**-**^/CD19^**-**^/CD45^**-**^/F4/80^**-**^) in lungs of naive and allergic mice was investigated by flow cytometry. Data are the pool from three independently performed experiments of identical design. Values represent means ± SEM. A value P<0.05 was considered to be significant. *P<0.05, **P<0.01 and ***P<0.001 indicate levels significantly different between naive and allergic mice. ATII-LCs = ATII-like cells; DCs = dendritic cells; Mϕ = macrophages.

Bv1-Peptide-FAM and Bv1-Protein-FAM were intranasally applied to either naive or Bet v 1-sensitised and birch pollen-challenged mice (Fig 2A). One hour after Bv1-Peptide application or six hours after Bv1-Protein application, phenotypic characterisation of FAM^+^ cells in lungs was performed (Fig 2B and 2C). ATII-LCs were the major cell population in uptake of both antigens in naive mice. However, in sensitised and challenged mice, macrophages displayed the dominant cell population internalising either Bv1-Protein or Bv1-Peptide.

### After intranasal application, Bv1-Peptide is taken up at an earlier time point and more efficiently by cells of the NALT and lungs, compared to Bv1-Protein

**Fig 3:**
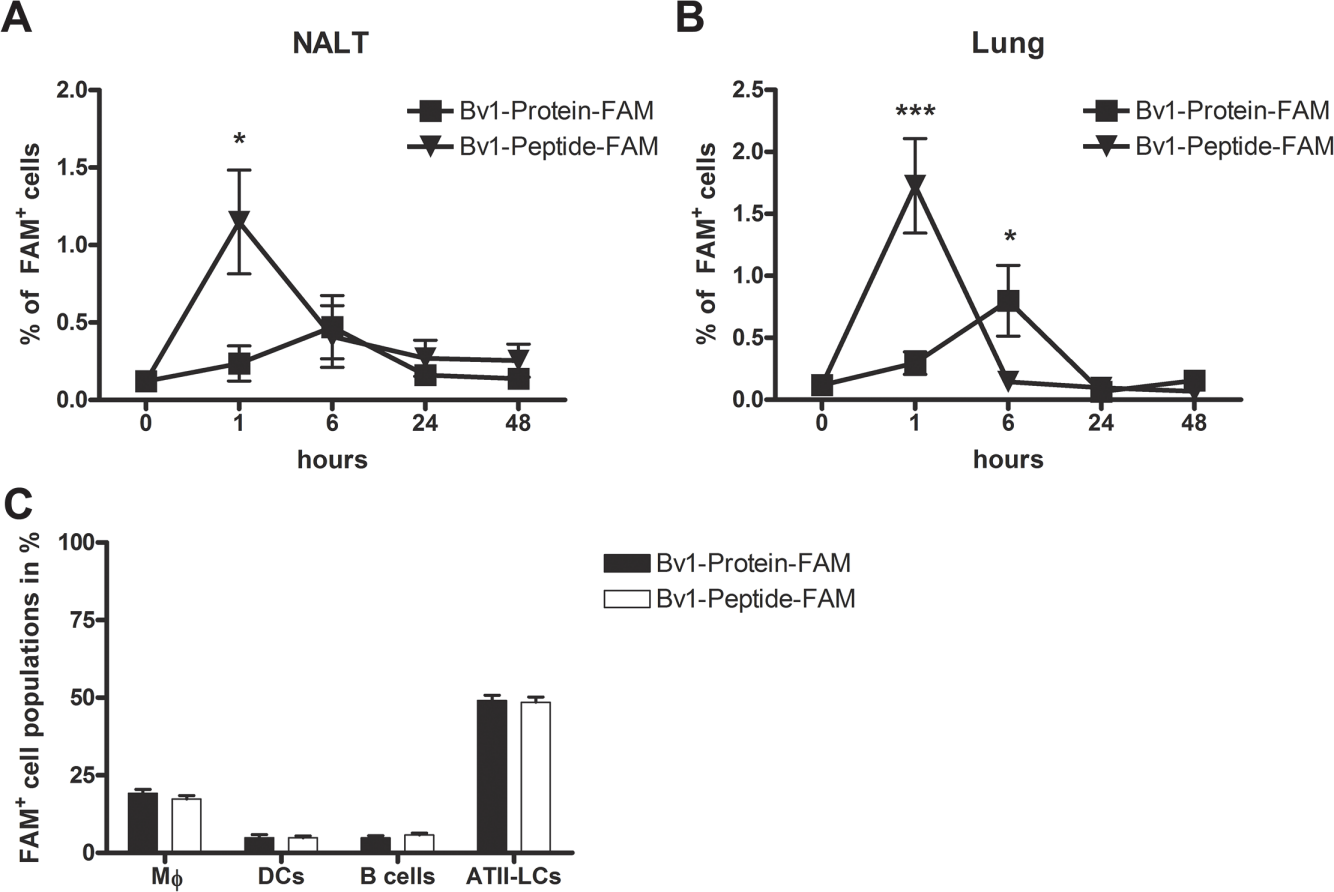
Time-dependent uptake of Bv1-Protein and Bv1-Peptide in NALT and lungs and phenotypic characterisation of FAM^+^ lung celIs *in vivo*. 20 μg of Bv1-Protein-FAM or Bv1-Peptide-FAM were intranasal administered to naive BALB/c mice (n = 3 per time point). (**A** and **B**) After 1, 6, 24, and 48 hours NALT and lungs were harvested, and analysed by flow cytometry. Dead cells were identified via 7-AAD staining and excluded from analysis. Data are the pool from three independently performed experiments of identical design. (**C**) FAM^**+**^ cells were gated and antigen uptake capacity of macrophages (CD11b^**+**^/CD11c^**-**^), dendritic cells (CD11b^**-**^/CD11c^**+**^), B cells (B220^**+**^/CD19^**+**^), and ATII-LCs (CD11b^**-**^/CD11c^**-**^/CD16/32^**-**^/CD19^**-**^/CD31^**-**^/CD45^**-**^/F4/80^**-**^/MHCII^**+**^) in lungs of naive mice was investigated by flow cytometry. Data are the pool of two independently performed experiments of identical design. Values represent means ± SEM. A value P<0.05 was considered to be significant. *P<0.05 and ***P<0.001 indicate levels significantly different from time point 0 hours. ATII-LCs = ATII-like cells; DCs = dendritic cells; Mϕ = macrophages; NALT = nasal associated lymphoid tissue.

In order to investigate antigen uptake *in vivo*, Bv1-Protein and Bv1-Peptide were intranasally applied to naive BALB/c mice and cells of the NALT and lungs were collected. The number of Bv1-Peptide-FAM^+^ cells reached highest levels already after 1 hour after *in vivo* application in both NALT and lungs. In contrast, Bv1-Protein internalisation peaked 6 hours after intranasal application (Fig 3A and 3B). After 24 hours none of the antigens were detectable in NALT or lung cells. Phenotypic characterisation of the lung cells revealed that antigens were taken up preferably by ATII-LCs, followed by macrophages, DCs and B cells (Fig 3C). In NALT, majority of both antigens was taken up by B cells (S1 Fig). There were no FAM-positive DCs and macrophages in NALT (S1 Fig). Precise gating strategy is displayed in S1 and S2 Figs.

### A549 cells internalised Bv1-Peptide earlier and more efficiently than Bv1-Protein *in vitro*

**Fig 4:**
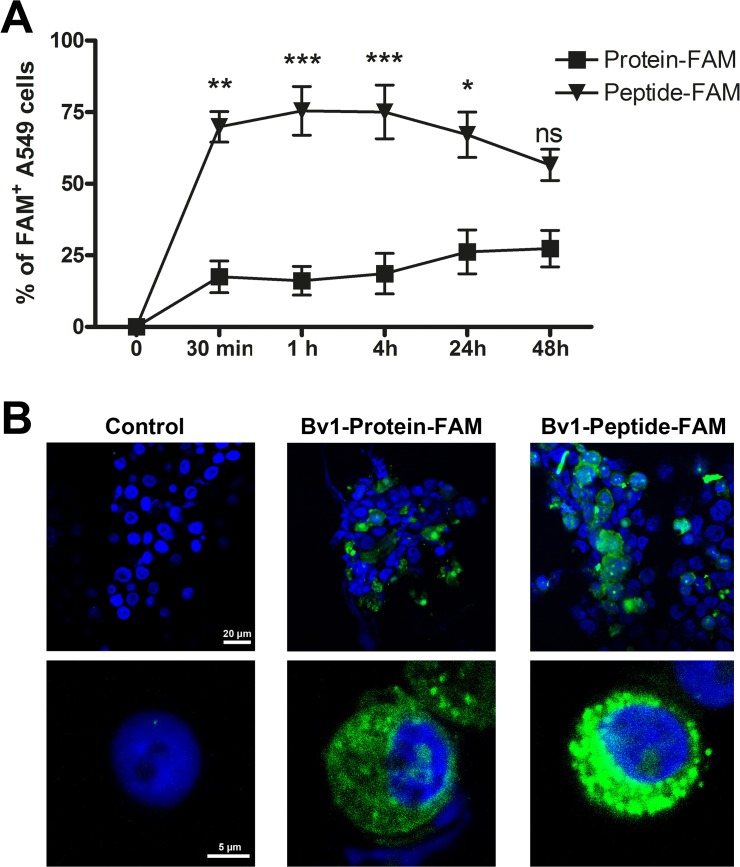
Uptake of Bv1-Protein and Bv1-Peptide in A549 cells. The human ATII cell line A549 was incubated with 5 μg/ml of Bv1-Protein-FAM and Bv1-Peptide-FAM for 0.5, 1, 4, 24, and 48 hours. (**A**) The uptake was measured via flow cytometry. Dead cells were identified via 7-AAD staining and excluded from analysis. Data are the pool from three independently performed experiments of identical design. Values represent means ± SEM. A value P<0.05 was considered to be significant. *P<0.05, **P<0.01 and ***P<0.001 indicate levels significantly different between A549 cells stimulated with Bv1-Protein or Bv1-Peptide. (**B**) A549 cells were cultured for 4 hours with Bv1-Protein-FAM and Bv1-Peptide-FAM (green), transferred on glass slides, stained and mounted. Examination was performed with a confocal microscope. Nuclear counterstain was performed with DAPI (blue). ns = not significant.

**Fig 5:**
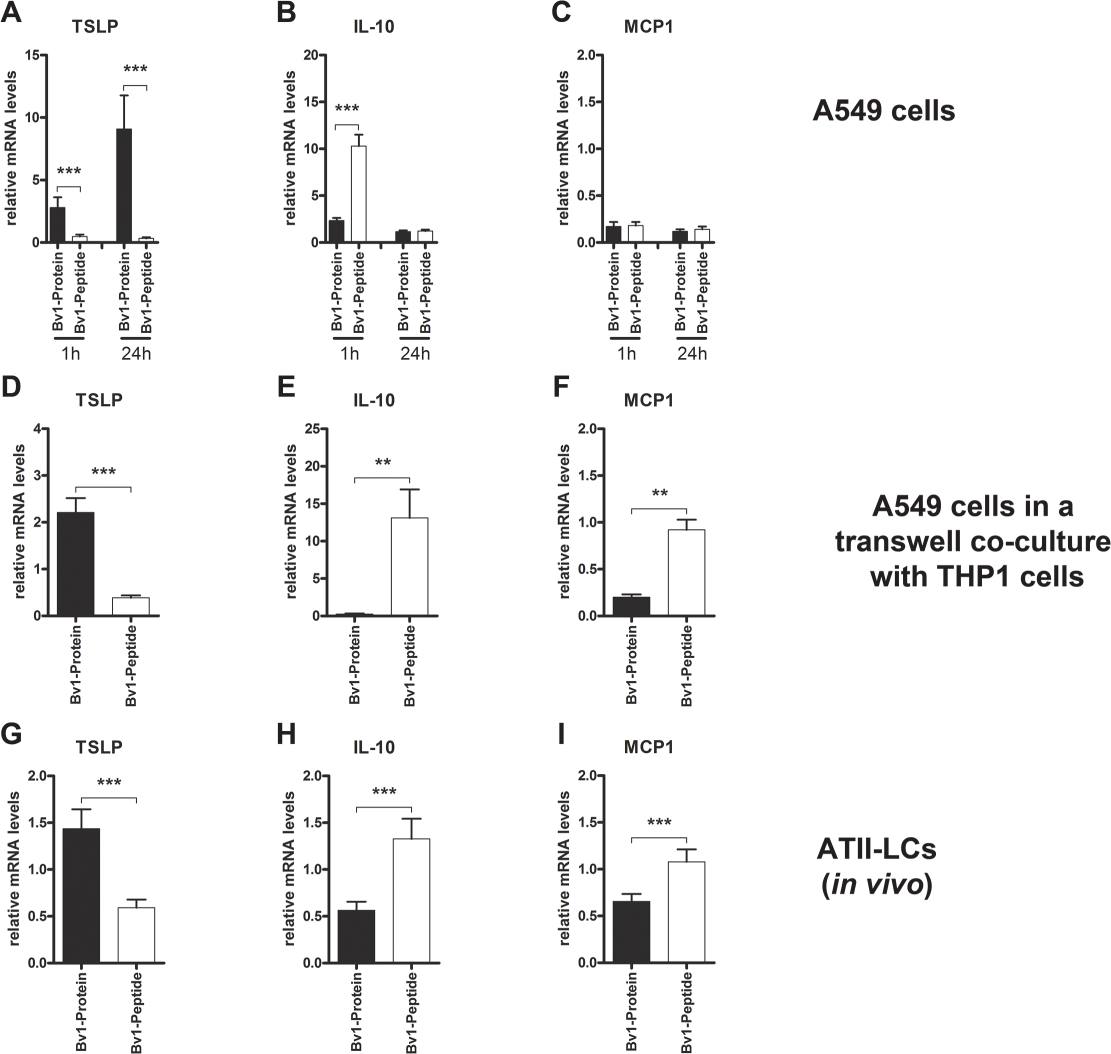
Bv1-Protein markedly increases levels of TSLP mRNA, whereas Bv1-Peptide induces IL-10 and MCP1 mRNA transcription. (**A-C**) A549 cells (5x10^**5**^ cells/well) were stimulated with 5 μg/ml of Bv1-Protein or Bv1-Peptide for 1 and 24 hours. A549 cells were harvested to evaluate gene expression. Reactions were executed in triplicates and mRNA gene expression of TSLP, IL-10 and MCP1 in A549 cells was normalised to the expression of the housekeeping gene β-actin and relative gene quantification was performed by comparing RNA samples at 1 and 24 hour intervals to control samples at 0 hours. Data are the pool from three independently performed experiments of identical design. (**D-F**) A549 cells (5x10^**5**^ cells/well) were co-cultured in a transwell system with THP1 cells (5x10^**5**^ cell/well) and stimulated with 5 μg/ml of Bv1-Protein and Bv1-Peptide for 6 days. A549 and THP1 cells were harvested separately to evaluate gene expression. Reactions were executed in triplicates and mRNA gene expression of TSLP, IL-10 and MCP1 in A549 cells was normalised to the expression of the housekeeping gen β-actin and relative gene quantification was performed by comparing RNA samples of stimulated cells to control samples. Data are the pool from three independently performed experiments of identical design. (**G-I**) 20 μg of Bv1-Protein or Bv1-Peptide were intranasal administered to naive BALB/c mice (n = 3 per time point). Primary ATII-LCs were isolated from lungs 24 hours after intranasal application to evaluate gene expression. Reactions were executed in triplicates and mRNA gene expression of TSLP, IL-10 and MCP1 in ATII-LCs was normalised to the expression of the housekeeping gene β-actin and relative gene quantification was performed by comparing RNA samples of stimulated cells to control samples. Data are the pool from two independently performed experiments of identical design. (**A-I**) Values represent means ± SEM. A value P<0.05 was considered to be significant. **P<0.01 and ***P<0.001 indicate levels significantly different between A549 or ATII-LCs stimulated with Bv1-Protein and Bv1-Peptide. ATII-LCs = ATII-like cells; MCP1 = Monocyte chemoattractant protein-1; TSLP = Thymic stromal lymphopoietin.

Already 30 minutes after incubation of the human ATII cell line, A549, with Bv1-Peptide-FAM, 70% of all cells were FAM^+^. Even after 48 hours, 50% of cells remained positive. In contrast, after 30 minutes of incubation with Bv1-Protein-FAM, only 20% were FAM^+^. This number did not significantly increase over the experimental period of 48 hours (Fig 4A). Internalisation of both antigens after 4 hours was confirmed by confocal microscopy (Fig 4B). Incubation of A549 cells with Bv1-Protein led to increased levels of TSLP mRNA (Fig 5A), while Bv1-Peptide induced the transcription of IL-10 mRNA (Fig 5B). Levels of MCP1 mRNA remained unchanged after 24 hour incubation with Bv1-Protein or Bv1-Peptide (Fig 5C).

In a transwell co-culture system, A549 and THP1 cells were stimulated with Bv1-Protein and Bv1-Peptide. The gene expression of both cell types was evaluated separately after 6 days. A549 show significant elevated levels of MCP1 and IL-10 upon stimulation with Bv1-Peptide, whereas incubation with Bv1-Protein increases mRNA expression of TSLP (Fig 5D–5F). No induction of IL-10, TSLP or MCP1 mRNA was found in THP1 cells after incubation with both antigens (data not shown).

Primary murine ATII-LCs, which were isolated from mice after intranasal application of Bv1-Peptide, reveal significant levels of IL-10 and MCP1 mRNA (Fig 5H and 5I), whereas Bv1-Protein induced transcription of TSLP mRNA (Fig 5G) in these cells. These data are in accordance with data obtained from transwell co-culture experimentswith A549 and THP1 cell lines (Fig 5D–5F).

### *In vitro* incubation of A549/THP1 co-culture with Bv1-Peptide induced significantly higher levels of IL-6 and IL-8 than incubation with Bv1-Protein

**Fig 6:**
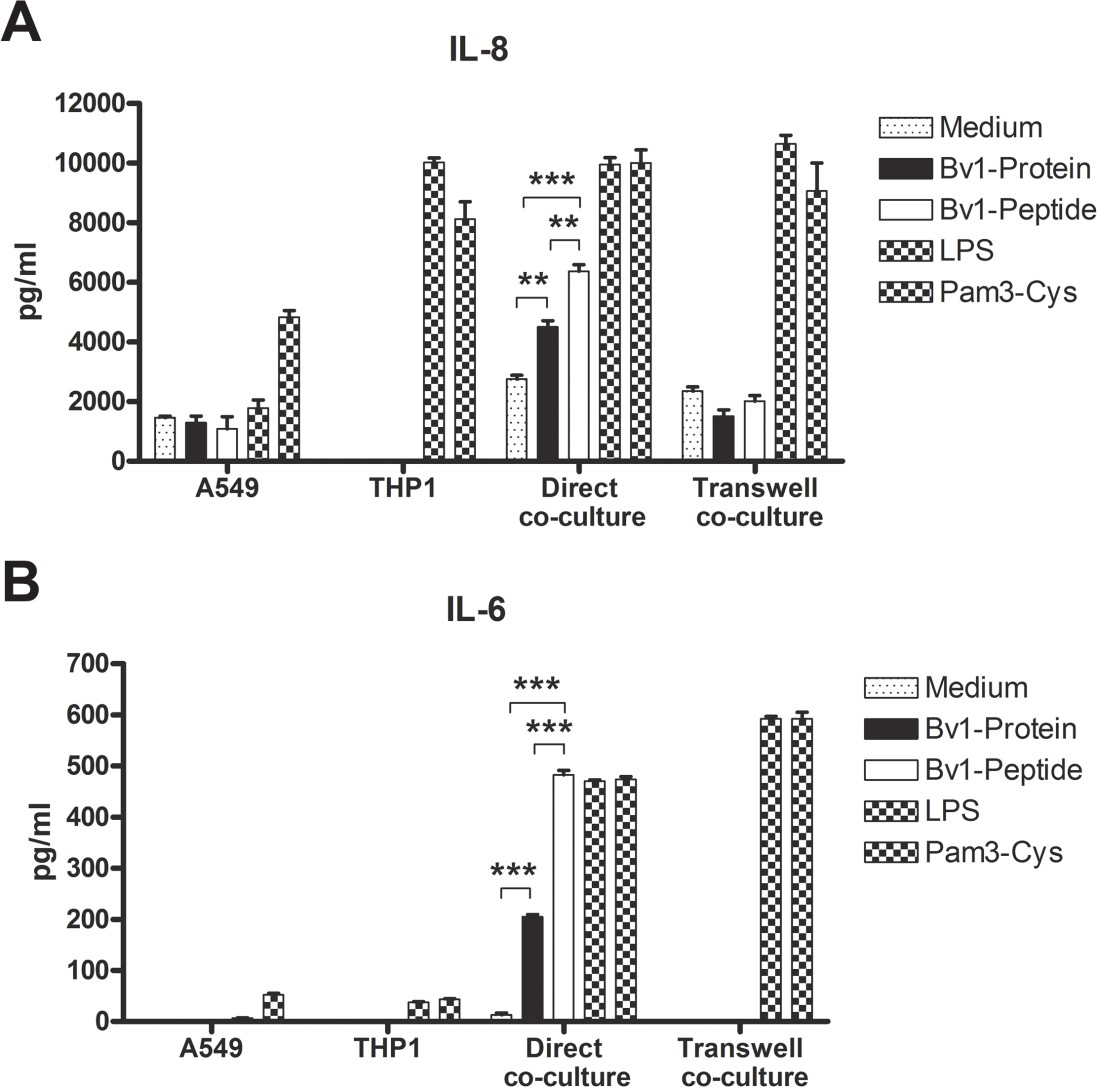
Direct and transwell co-culture of A549 and THP1. A549 cells (5x10^**5**^ cells/well) were co-cultured with THP1 cells (5x10^**5**^ cell/well) directly or in a transwell system and stimulated with 5 μg/ml of Bv1-Protein and Bv1-Peptide or with 1 μg of LPS or 1 μg of Pam3-Cys for 6 days. IL-8 (**A**) and IL-6 (**B**) levels were measured in cell culture supernatants using ELISA. Values represent means ± SEM. A value P<0.05 was considered to be significant. **P<0.01 and ***P<0.001.

Without any stimulus, A549 cells constitutively secrete IL-8. Production of the cytokine was increased by Pam3-Cys, but was not altered by addition of Bv1-Protein, Bv1-Peptide, or LPS (Fig 6A). In THP1 cells, both LPS and Pam3-Cys induced high levels of IL-8, but level of this cytokine was under detection limit in cultures incubated with Bv1-Protein and Bv1-Peptide (Fig 6A). Furthermore, neither A549 nor THP1 cells produce IL-6 after incubation with Bv1-Protein or Bv1-Peptide (Fig 6B). In contrast, direct co-culture of these two cell lines led to increased levels of IL-6 and IL-8 upon addition of Bv1-Protein or Bv1-Peptide (Fig 6). Moreover, Bv1-Peptide induced significantly higher levels of both cytokines compared to Bv1-Protein. This cytokine production was depended on direct cell contact, as no differences in cytokine production were detected in transwell culture system (Fig 6).

## Discussion

In the present study we sought to investigate, whether the conformation/size of molecules used for intranasal tolerance induction impacts on uptake by host cells and polarisation of the immune responses. Therefore, internalisation of two different allergen molecules, the three-dimensional Bv1-Protein and the linear Bv1-Peptide, was investigated in NALT and lungs, as primary sites of antigen uptake in the respiratory mucosa.

*In vitro* studies with lung cells revealed that both Bv1-Peptide and Bv1-Protein were taken up with the same intensity at all investigated time points (maximum uptake was detected after 6 hours, Fig 1), where around 15–20% of all cells were FAM^+^. In contrast, investigation of antigen uptake *in vivo*, after intranasal application, showed that Bv1-Peptide was internalised earlier and more efficiently than Bv1-Protein in NALT and lungs (Fig 3), however, only around 1–2% of all cells were FAM^+^ in the case of Bv1-Peptide and only around 0.5% in the case of Bv1-Protein. Moreover, Bv1-Peptide was internalised faster and at an earlier time point compared to Bv1-Protein. These data indicated that the size of the antigens might be an important factor for the effectivity and kinetic of the uptake. Accordingly, several studies investigated the relationship between increasing molecular weight and the effectivity of transport across the mucosal membrane in the respiratory tract and concluded an inverse relationship between molecule size and amount of antigen uptake [23,24]. Moreover, we observed differences in intensity of uptake between *in vitro* and *in vivo* conditions. The nasal respiratory epithelia is covered with a constantly regenerating mucus, responsible for the fact that nasally-applied molecules are cleared from the mucosa within a short period (i.e. around 20 minutes) [23]. Thus, there is a narrow “window of opportunity” for antigens to be available for mucosal cells in comparison to *in vitro* conditions, where antigens are in the direct contact with cell populations of the respiratory tract during the incubation period (i.e. 0.5–48 hours).

Based on our kinetic studies on antigen uptake *in vitro* (Fig 1), phenotypic characterisation of lung cells taking up either Bv1-Protein or Bv1-Peptide was performed. Here we describe that in naive mice, ATII-LCs are the major population internalising both Bv1-Protein and Bv1-Peptide *in vitro* and *in vivo* (Figs 1 and 3). Apart from the fact that dendritic cells are described as a prominent population responsible for antigen uptake in airways [25,26], our data on ATII-LCs are supported also by other studies [27,28]. Moreover, these authors showed that small and larger molecules (<2 kDa and >60 kDa) diffuse across airways and are taken up and captured for several hours in alveolar epithelial cells [27,28].

Surprisingly, we noted a marked difference in cell types internalising antigens in allergic versus naive mice, where macrophages, but not ATII-LCs, were the dominant population internalising Bv1-Protein and Bv1-Peptide (>50% of FAM^+^ cells were macrophages in allergic mice, while >50% of FAM^+^ cells were ATII-LCs in naive mice). In a mouse model of birch pollen allergy, we previously described that airway challenge with birch pollen leads to increased recruitment of macrophages into the alveolar space [5]. Accordingly, the important role of macrophages for allergen uptake in an allergic status has been demonstrated in a mouse model of OVA-induced airway inflammation [29]. An increased allergen uptake was associated with increased secretion of surfactant proteins by ATII cells [29]. Thus, we suggest that the immune status of the host might be another important factor influencing the process of antigen uptake by distinct cell populations after intranasal application.

As our data pointed out the importance of ATII-LCs in the antigen uptake process, further studies on the functionality of these cells were performed. Previous studies using isolated murine ATII cells showed their trans-differentiation towards ATI cells within days during *in vitro* cultivation [30,31], indicating that they are not suitable for long term experiments *in vitro*. We therefore used the human ATII cell line A549 for further experiments. When stimulating A549 cells alone or in a transwell co-culture with the monocyte cell line THP1 with Bv1-Protein, enhanced TSLP mRNA levels, known to initiate or enhance T_H_2 responses at the lung epithelial cell surface [32], were measured in A549 cells, whereas Bv1-Peptide induced the transcription of IL-10 mRNA (Fig 5). Our data indicate a distinct polarisation pattern of A549 cells depending on the encountered antigen. Similar to our observations, it has been shown that stimulation with correctly folded allergens, such as phospholipase A_2_ or OVA, induces T_H_2 immune responses, while structurally modified allergens (e.g. heat-treated OVA) rather induce T_H_1 responses [33,34]. The capacity of peptides to induce IL-10 as described in our study is also supported by other investigators, suggesting allergen peptides as suitable candidates for tolerance induction (33).

Beside their barrier function, ATII cells are able to produce several chemokines, important for the recruitment of monocytes and lymphocytes, of which MCP1 is a representative candidate [35,36]. While stimulation of A549 cells alone with allergen molecules did not induce upregulation of this chemokine, A549 cells in a transwell co-culture with monocytes (THP1) led to increased MCP1 mRNA transcription upon Bv1-Peptide, but not Bv1-Protein, stimulation (Fig 5). These data indicate that A549 cells are able to crosstalk with other neighbouring cells, such as monocytes/macrophages, in an antigen-specific but cell-cell contact-independent manner.

Importantly, the analysis of TSLP, IL-10 and MCP1 mRNA transcription in primary ATII-LCs isolated from naive mice 6 hours after the intranasal application of Bv1-Protein or one hour after the intranasal application of Bv1-Peptide revealed similar results compared to the A549 and THP1 co-culture (Fig 5). When performing direct co-culture of A549 cells with THP1 cells, we measured increased levels of IL-6 and IL-8 after incubation with Bv1-Protein and Bv1-Peptide (Fig 6), similarly as previously described by Striz *et al*. 2001 [37]. In order to investigate whether cell-cell contact between A549 and THP1 cells is important for cytokine production, transwell co-culture was performed (Fig 6), showing that indeed direct cell-cell contact is necessary for production of these cytokines.

In conclusion, our findings emphasise the importance of ATII-LCs in the uptake of antigens entering via the respiratory mucosa. This study further indicates that ATII-LCs may contribute to polarisation processes of the immune system depending on the structure and size of the encountered antigen during the non-inflamed steady state situation. Further investigations on the role and function of these cells during health and allergic disease may help to improve prophylactic as well as therapeutic strategies against asthma and allergic diseases.

## Supporting Information

S1 Fig Gating strategy for NALT. NALT cells were harvested and analysed by flow cytometry. Dead cells were identified via 7-AAD staining and excluded from analysis (Step 1). FAM^+^ cells were gated (Step 2) and phenotypic cell characterisation of FAM^+^ cells was performed using specific markers for macrophages (CD11b^+^/CD11c^-^), dendritic cells (CD11b^-^/CD11c^+^) and B cells (B220^+^/CD19^+^) (Step 3). NALT = nasal associated lymphoid tissue.
(TIF)

S2 Fig Gating strategy for the lung. Lung cells were harvested and analysed by flow cytometry. Dead cells were identified via 7-AAD staining and excluded from analysis (Step 1). FAM^+^ cells were gated (Step 2) and phenotypic cell characterisation of FAM^+^ cells was performed using specific markers for macrophages (CD11b^+^/CD11c^-^), dendritic cells (CD11b^-^/CD11c^+^), B cells (B220^+^/CD19^+^), and ATII-LCs (CD11b^-^/CD11c^-^/CD16/32^-^/CD19^-^/CD31^-^/CD45^-^/F4/80^-^/MHCII^+^) (Step 3). ATII-LCs = ATII-like cells.
(TIF)
