# Supplementary figures and images for: The Role of Alveolar Epithelial Type II-Like Cells in Uptake of Structurally Different Antigens and in Polarisation of Local Immune Responses
Source: PLoS One. 2015 Apr 20;10(4):e0124777. doi: 10.1371/journal.pone.0124777 (PMC4404363; doi:10.1371/journal.pone.0124777)

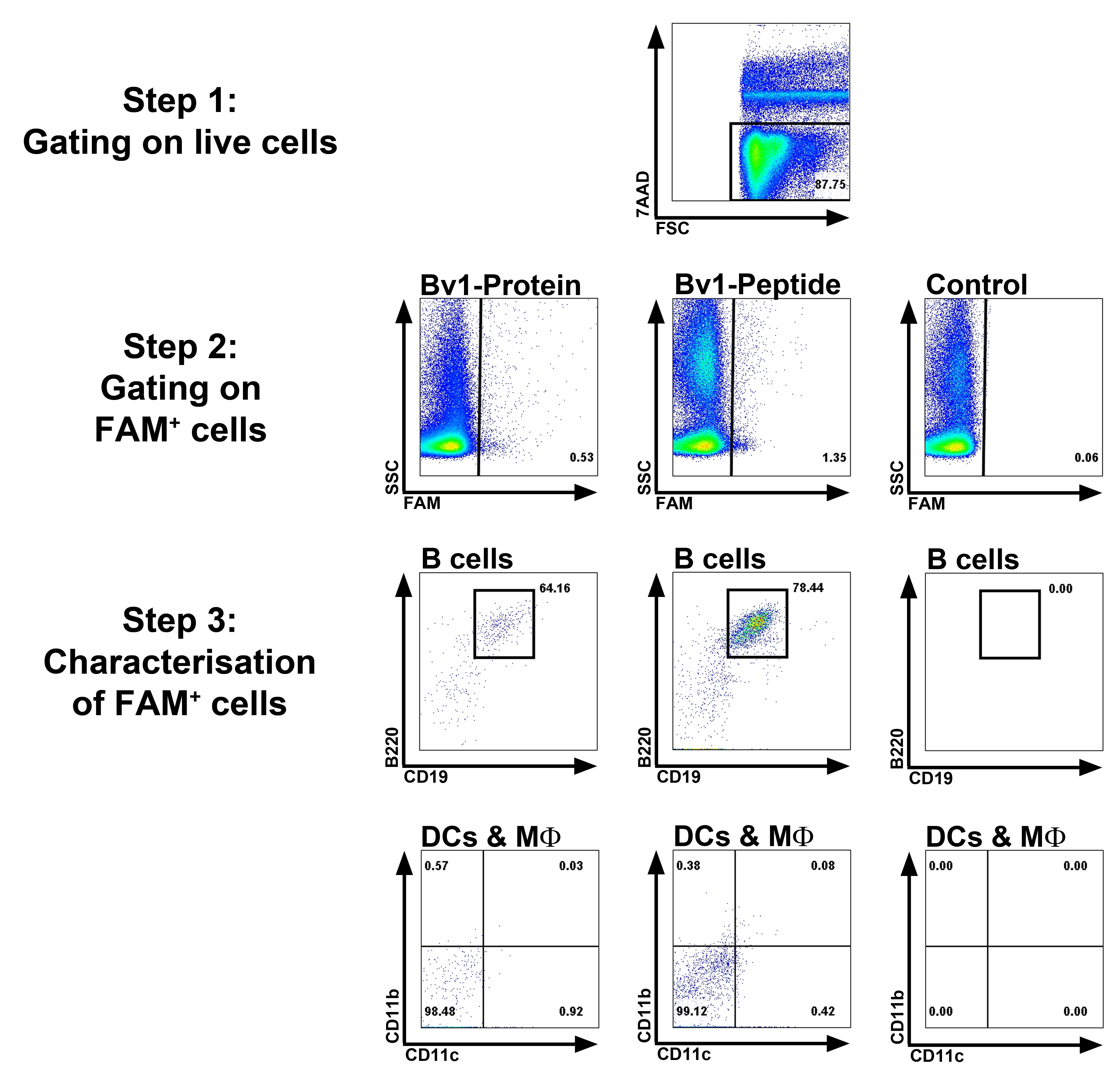

Supplement: S1 Fig — NALT cells were harvested and analysed by flow cytometry. Dead cells were identified via 7-AAD staining and excluded from analysis (Step 1). FAM+ cells were gated (Step 2) and phenotypic cell characterisation of FAM+ cells was performed using specific markers for macrophages (CD11b+/CD11c-), dendritic cells (CD11b-/CD11c+) and B cells (B220+/CD19+) (Step 3). NALT = nasal associated lymphoid tissue. (TIF) [file pone.0124777.s001.tif]

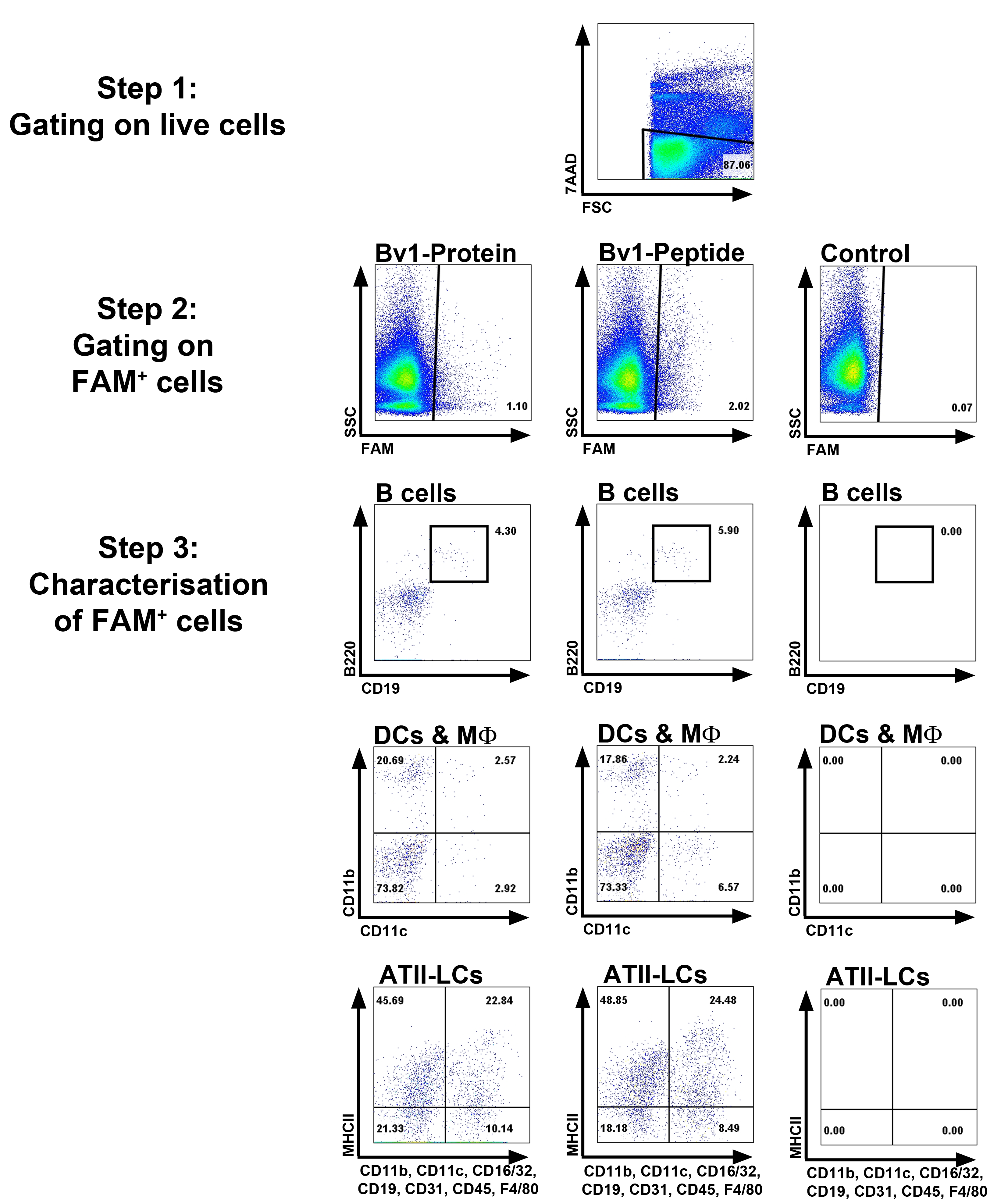

Supplement: S2 Fig — Lung cells were harvested and analysed by flow cytometry. Dead cells were identified via 7-AAD staining and excluded from analysis (Step 1). FAM+ cells were gated (Step 2) and phenotypic cell characterisation of FAM+ cells was performed using specific markers for macrophages (CD11b+/CD11c-), dendritic cells (CD11b-/CD11c+), B cells (B220+/CD19+), and ATII-LCs (CD11b-/CD11c-/CD16/32-/CD19-/CD31-/CD45-/F4/80-/MHCII+) (Step 3). ATII-LCs = ATII-like cells. (TIF) [file pone.0124777.s002.tif]
